# Supplementary material for: Developmental oligodendrocytes regulate brain function through the mediation of synchronized spontaneous activity
Source: eLife. 2026 Jun 18;14:RP102200. doi: 10.7554/eLife.102200 (PMC13278736; doi:10.7554/eLife.102200)
Supplement: Peer review history

# Editor report: eLife Assessment

**DOI:** 10.7554/eLife.102200.4.sa0

**Authors:** K VijayRaghavan

**Affiliations:** National Centre for Biological Sciences, Tata Institute of Fundamental Research India

**Keywords:** Compelling, Important

This study investigates the role of developmental oligodendrocytes in synchronising spontaneous activity in neuronal circuits and influencing cerebellar-dependent behaviour. The authors use advanced viral targeting techniques to deplete oligodendrocytes in a cell-specific manner, paired with in vivo calcium imaging of Purkinje cells, to establish a relationship between oligodendrocyte-mediated neuronal synchrony and complex brain function. The authors present **compelling** evidence of oligodendrocyte-regulated neuronal synchrony. Overall, this manuscript holds promise as an **important** contribution to neurodevelopment research.

# Referee report: Reviewer #1 (Public review)

**DOI:** 10.7554/eLife.102200.4.sa1

[Editor's note: this version has been assessed by the Reviewing Editor without further input from the original reviewers. The authors have satisfactorily addressed the previous concerns raised by the reviewers.]

Summary:

This study presents convincing findings that oligodendrocytes play a regulatory role in spontaneous neural activity synchronization during early postnatal development, with implications for adult brain function. Utilizing targeted genetic approaches, the authors demonstrate how oligodendrocyte depletion impacts Purkinje cell activity and behaviors dependent on cerebellar function. Delayed myelination during critical developmental windows is linked to persistent alterations in neural circuit function, underscoring the lasting impact of oligodendrocyte activity.

Strengths:

(1) The research leverages the anatomically distinct olivocerebellar circuit, a well-characterized system with known developmental timelines and inputs, strengthening the link between oligodendrocyte function and neural synchronization.

(2) Functional assessments, supported by behavioral tests, validate the findings of in vivo calcium imaging, enhancing the study's credibility.

(3) Extending the study to assess long-term effects of early life myelination disruptions adds depth to the implications for both circuit function and behavior.

Weaknesses:

(1) The study would benefit from a closer analysis of myelination during the periods when synchrony is recorded. Direct correlations between myelination and synchronized activity would substantiate the mechanistic link and clarify if observed behavioral deficits stem from altered myelination timing.

(2) Although the study focuses on Purkinje cells in the cerebellum, neural synchrony typically involves cross-regional interactions. Expanding the discussion on how localized Purkinje synchrony affects broader behaviors-such as anxiety, motor function, and sociality - would enhance the findings' functional significance.

(3) The authors discuss the possibility of oligodendrocyte-mediated synapse elimination as a possible mechanism behind their findings, drawing from relevant recent literature on oligodendrocyte precursor cells. However, there are no data presented supporting these assumptions. The authors should explain why they think the mechanism behind their observation extends beyond the contribution of myelination or remove this point from the discussion entirely.

Comment for resubmission: Although the argument on synaptic elimination has been removed, it has been replaced with similarly unclear speculation about roles for oligodendrocytes outside of conventional myelination or metabolic support, again without clear evidence. The authors measured MBP area but have not performed detailed analysis of oligodendrocyte biology to support the claims made in the discussion. Please consider removing this section or rephrasing it to align with the data presented.

(4) It would be valuable to investigate secondary effects of oligodendrocyte depletion on other glial cells, particularly astrocytes or microglia, which could influence long-term behavioral outcomes. Identifying whether the lasting effects stem from developmental oligodendrocyte function alone or also involve myelination could deepen the study's insights.

(5) The authors should explore the use of different methods to disturb myelin production for a longer time, in order to further determine if the observed effects are transient or if they could have longer-lasting effects.

(6) Throughout the paper, there are concerns about statistical analyses, particularly on the use of the Mann-Whitney test or using fields of view as biological replicates.

# Author comment: Author response

**DOI:** 10.7554/eLife.102200.4.sa2

**Authors:** Ryo Masumura, Kyosuke Goda, Mariko Sekiguchi, Naofumi Uesaka

**Affiliations:** Institute of Science Tokyo Tokyo Japan; Tokyo Medical and Dental University Tokyo Japan; Tokyo Medical and Dental University Tokyo Japan; Institute of Science Tokyo Tokyo Japan

The following is the authors’ response to the previous reviews

**Public Reviews:**

**Reviewer #1 (Public review):**

Summary:

This study presents convincing findings that oligodendrocytes play a regulatory role in spontaneous neural activity synchronisation during early postnatal development, with implications for adult brain function. Utilising targeted genetic approaches, the authors demonstrate how oligodendrocyte depletion impacts Purkinje cell activity and behaviours dependent on cerebellar function. Delayed myelination during critical developmental windows is linked to persistent alterations in neural circuit function, underscoring the lasting impact of oligodendrocyte activity.

Strengths:

(1) The research leverages the anatomically distinct olivocerebellar circuit, a well-characterized system with known developmental timelines and inputs, strengthening the link between oligodendrocyte function and neural synchronization.

(2) Functional assessments, supported by behavioral tests, validate the findings of in vivo calcium imaging, enhancing the study's credibility.

(3) Extending the study to assess the long-term effects of early-life myelination disruptions adds depth to the implications for both circuit function and behavior.

We appreciate these positive evaluation.

Weaknesses:

(1) The study would benefit from a closer analysis of myelination during the periods when synchrony is recorded. Direct correlations between myelination and synchronized activity would substantiate the mechanistic link and clarify if observed behavioral deficits stem from altered myelination timing.

We appreciate the reviewer’s thoughtful suggestion and have expanded the manuscript to clarify how oligodendrocyte maturation relates to the development of Purkinje-cell synchrony. The developmental trajectory of Purkinje-cell synchrony has already been comprehensively characterized by Good et al. (2017, Cell Reports 21: 2066–2073): synchrony drops from a high level at P3–P5 to adult-like values by P8. We found that the myelination in the cerebellum starts to appear from P5-P7 (Figure S1A, B), indicating that the timing of Purkinje cell desynchronization coincides with the initial appearance of oligodendrocytes and myelin in the cerebellum. To determine whether myelin growth could nevertheless modulate this process, we quantified ASPA-positive oligodendrocyte density and MBP-positive bundle thickness and area at P10, P14, P21 and adulthood (Fig. 1J, K, Fig. S1E). Both metrics increase monotonically and clearly lag behind the rapid drop in synchrony, indicating that myelination could be not the primary trigger for the desynchronization. When oligodendrocytes were ablated during the second postnatal week, the synchrony was reduced (new Fig. 2). Thus, once myelination is underway, oligodendrocytes become critical for maintaining the synchrony, acting not as the initiators but as the stabilizers and refiners of the mature network state.

We have added the new subsection in discussion (lines 451–467) now in which we propose a two-phase model. Phase I (P3–P8): High early synchrony is generated by non-myelin mechanisms (e.g. transient gap junctions, shared climbing-fiber input). Phase II (P8-). As oligodendrocytes proliferate and ensheath axons, they fine-tune conduction velocity and stabilize the mature, low-synchrony network state.

We believe these additions fully address the reviewer’s concerns.

(2) Although the study focuses on Purkinje cells in the cerebellum, neural synchrony typically involves cross-regional interactions. Expanding the discussion on how localized Purkinje synchrony affects broader behaviors - such as anxiety, motor function, and sociality - would enhance the findings' functional significance.

We appreciate the reviewer’s helpful suggestion and have expanded the Discussion (lines 543–564) to clarify how localized Purkinje-cell synchrony can influence broader behavioral domains. In the revised text we note that changes in PC synchrony propagate into thalamic, prefrontal, limbic, and parietal targets, thereby impacting distributed networks involved in motor coordination, affect, and social interaction. Our optogenetic rescue experiments further support this framework, as transient resynchronization of PCs normalized sociability and motor coordination while leaving anxiety-like behavior impaired. This dissociation highlights that different behavioral domains rely to varying degrees on precise cerebellar synchrony and underscores how even localized perturbations in Purkinje timing can acquire system-level significance.

(3) The authors discuss the possibility of oligodendrocyte-mediated synapse elimination as a possible mechanism behind their findings, drawing from relevant recent literature on oligodendrocyte precursor cells. However, there are no data presented supporting this assumption. The authors should explain why they think the mechanism behind their observation extends beyond the contribution of myelination or remove this point from the discussion entirely.

We thank the reviewer for pointing out that our original discussion of oligodendrocyte-mediated synapse elimination was not directly supported by data in the present manuscript. Because we are actively analyzing this question in a separate, follow-up study, we have deleted the speculative passage to keep the current paper focused on the demonstrated, myelination-dependent effects. We believe this change sharpens the mechanistic narrative and fully addresses the reviewer’s concern.

(4) It would be valuable to investigate the secondary effects of oligodendrocyte depletion on other glial cells, particularly astrocytes or microglia, which could influence long-term behavioral outcomes. Identifying whether the lasting effects stem from developmental oligodendrocyte function alone or also involve myelination could deepen the study's insights.

We thank the reviewer for raising this point and have performed the requested analyses. Using IBA1 immunostaining for microglia and S100b for Bergmann glia, we quantified cell density and these marker signal intensity at P14 and P21. Neither microglial or Bergmann-glial differed between control and oligodendrocyte-ablated mice at either time‐point (new Figure S2). These results indicate that the behavioral phenotypes we report are unlikely to arise from secondary activation or loss of other glial populations.

We now added results (lines 275–286) and also discuss myelination and other oligodendrocyte function (lines 443–450). It remains difficult to disentangle conduction-related effects from myelination-independent trophic roles of oligodendrocytes. We therefore note explicitly that future work employing stage-specific genetic tools or acute metabolic manipulations will be required to parse these contributions more definitively.

(5) The authors should explore the use of different methods to disturb myelin production for a longer time, in order to further determine if the observed effects are transient or if they could have longer-lasting effects.

We agree that distinguishing transient from enduring effects is critical. Importantly, our original submission already included data demonstrating a persistent deficit of PC population synchrony (Fig. 4, previous Fig. 3): (i) at P14—the early age after oligodendrocyte ablation—population synchrony is reduced, and (ii) the same deficit is still present in adults (P60–P70) despite full recovery of ASPA-positive cell density and MBP-area and -thickness (Fig. 2H-K, Fig. S1E, and Fig. 4). We also performed the ablation of oligodendrocytes after the third postnatal week. Despite a similar acute drop in ASPA-positive cells, neither population synchrony nor anxiety-, motor-, or social behaviors differed from littermate controls. Thus, extending myelin disruption beyond the developmental window does not exacerbate or prolong the phenotype, whereas a short perturbation within that window leaves a permanent timing defect. These findings strengthen our conclusion that it is the developmental oligodendrocyte/myelination program itself—rather than ongoing adult myelin production—that is essential for establishing stable network synchrony. We now highlight this point explicitly in the revised Discussion (lines 507–522).

(6) Throughout the paper, there are concerns about statistical analyses, particularly on the use of the Mann-Whitney test or using fields of view as biological replicates.

We appreciate the reviewer’s guidance on appropriate statistical treatment. To address these concerns we have re-analyzed all datasets that contained multiple measurements per animal (e.g., fields of view, lobules, or trials) using nested statistics with animal as the higher-order unit. Specifically, we applied a two-level nested ANOVA when more than two groups were compared and a nested t-test when two conditions were present. The re-analysis confirmed all original conclusions. Because the nested models yielded comparable effect sizes to the Mann–Whitney tests, we have retained the mean ± SEM for ease of comparison with prior literature but now also report all values for each mouse in Table 1. In cases where a single measurement per mouse was compared between two groups, we used the Mann–Whitney test and present the results in the graphs as median values.
